# Evaluation of Options for Large Scalp Defect Reconstruction: A 12-Year Experience

**Published:** 2014-02-17

**Authors:** Dustin L. Eck, Stephanie L. Koonce, Bader M. Al Majed, Galen Perdikis

**Affiliations:** ^a^Department of General Surgery, Mayo Clinic, Jacksonville, Fla; ^b^Department of Plastic Surgery, Mayo Clinic, Jacksonville, Fla

**Keywords:** free flap, local tissue flap, microsurgery, scalp defects, scalp reconstruction

## Abstract

**Objective:** Multiple options for reconstruction of scalp defects exist with local tissue advancement and free tissue transfer the mainstay of reconstruction. Over the last 12 years, our tertiary referral hospital has performed more than 150 scalp reconstructions. We reviewed our experience with large scalp defects and evaluated whether free tissue transfer is a viable first option for reconstruction. **Methods:** A retrospective review was conducted of all scalp reconstructions from January 1, 1999, to December 31, 2011. A cohort of patients with defects greater than 50 cm^2^ were identified for a total of 64 operations; 10 free flaps, 28 local advancement flaps, and 26 skin grafts. Reoperation rates and complications were compared between groups. **Results:** Reoperation rate in the free flap group was 20% (2/10). Both reoperations were within the immediate postoperative period, one for microvascular thrombotic occlusion and the other for postoperative hematoma. The local tissue transfer group had a 14% reoperation rate (4/28), all for debridement of partial flap loss. The skin graft cohort had a 12% reoperation rate (3/26) for 1 complete and 2 partial skin graft failures; all required repeat grafting. Reoperation for free-flap complications did not require rehospitalization. In contrast, the skin graft and non–free flap reoperations frequently required rehospitalization. **Conclusion:** Though free tissue transfer has a higher occurrence of reoperation within the immediate postoperative period, completion of reconstruction usually occurs within a single hospitalization. Free tissue transfer is a feasible option, and we advocate for its use as a primary method for repairing large scalp defects.

Defects of the scalp arise from several diverse etiologies including trauma, burn injury, infection, radiation, surgical excision of tumor, or congenital lesion.^1^ Although smaller scalp defects may be closed primarily with simple undermining, larger defects may require advanced reconstruction approaches due to the relative inelasticity of surrounding tissue. Multiple options for reconstruction of large scalp defects exist. While historically the majority of larger defects required local flaps or skin grafts, advances in microsurgical techniques allowed free tissue flaps to emerge as an additional and possibly superior option for scalp reconstruction.^2^^-^5

We report our experience in reconstructing large defects (defined as >50 cm^2^) utilizing a variety of techniques including free flaps, local advancement flaps, and skin grafting.

## METHODS

A retrospective chart review was conducted of all patients who underwent scalp reconstruction for defects greater than 50 cm^2^ at our institution over a 12-year period, from January 1, 1999, to December 31, 2011. Patients with defects smaller than 50 cm^2^ or who were younger than 18 years were excluded. Data points included primary diagnosis, defect size, method of reconstruction, complications, and need for reoperations. The patients’ demographic data, medical history, surgical history, additional comorbidities, age, and gender were also analyzed.

## RESULTS

A total of 64 operations performed on 44 patients with large scalp defects were identified and included in the analysis. There were no significant differences between the 3 groups in age, sex, and comorbidities.

### Free flaps

Ten operations for scalp reconstruction using free vascularized tissue transfer were performed. Indications for surgery in these patients were squamous cell carcinoma (7/10), melanoma (1/10), spindle-cell fibroxanthoma tumor (1/10), and oligodendroma (1/10). The average defect size was 220 cm^2^ (range: 56–625 cm^2^). Four patients had undergone prior scalp reconstruction attempts; 1 with a local advancement flap and 3 with skin grafting. A latissimus dorsi free flap was used in 8 patients and rectus abdominis free flap in 2 patients. Two patients required a vein graft. The superficial temporal artery was used for all arterial anastomosis, and the superficial temporal vein was the recipient vessel in 8 patients, with the external jugular used in 2 patients. Two of the 10 free flap patients required reoperation (2/10; 20%). Of the patients requiring reoperations, 1 patient underwent emergent reexploration within the immediate postoperative period for microvascular thrombotic occlusion and the other for postoperative hematoma. Both patients completed their care within a single hospitalization and had complete integration of the flaps without further surgical intervention. Both patients requiring reoperation had undergone a previous scalp reconstruction procedure; one with a skin graft and the other with bilateral local advancement fasciocutaneous flaps.

### Local tissue transfer

Twenty-eight reconstructions with local tissue transfer flaps were performed. Two of the 28 patients had a previous reconstruction attempt. Prior treatments included 1 free tissue flap and 1 local advancement flap. Average defect size for the local flap group was 128 cm^2^ (range: 56–288 cm^2^). Ten patients had multiple local flaps during their operation. Four patients (14%; 4/28) underwent reoperations. Three patients required reoperation for debridement of partial flap loss and 1 patient for venous congestion with eventual complete revision of the flap. Of the 3 patients with partial flap loss, 1 patient needed only operative debridement, the second underwent delayed skin grafting, and the third patient required multiple operations with eventual need for a free tissue flap due to radiation induced necrosis of the local advancement flaps.

### Skin grafts

A total of 26 skin graft operations were performed. Ten of the 26 patients had a prior graft or flap reconstruction procedure: 3 free flaps, 3 local flaps, 1 skin graft, and 3 dermal substitute grafts. Average defect size for the skin graft cohort was 152 cm^2^ (range: 64–600 cm^2^) for patients solely undergoing skin graft and 161 cm^2^ (range: 15–625 cm^2^) for patients that had a concurrent flap reconstruction procedure. Three patients, 3 of 26 (11%) needed reoperation for graft failure: 1 for complete skin graft failure due to radiation and 2 partial skin graft failures. All required only repeat skin grafting.

## DISCUSSION

Several factors need to be considered when selecting the ideal flap for each individual scalp defect. The size of the defect, anatomic involvement, and overall health of the patient must all weigh in during the decision making process.^6^ Age alone is generally not a contraindication for scalp flap reconstruction. Both free tissue transfer and non–free tissue techniques are options for reconstruction of large scalp defects. With the relative inelasticity of scalp and forehead tissue, reconstruction of larger scalp defects with advancement flaps can be challenging. Even though free tissue transfer can have a higher occurrence of reoperation within the immediate postoperative period due to the complexity of the microvascular anastomosis, completion of reconstruction usually occurs within a single hospitalization. In addition, free tissue transfer does not require a staged reconstruction as seen in many local tissue transfer flaps.

Our rate of reoperation within the free flap group was not significantly different from the local advancement flap or skin graft groups, especially when eliminating reoperations for immediate postoperative failure. In the skin graft and non–free flap groups, the reoperations often required at least a second hospitalization, with some requiring multiple hospitalizations and procedures. No donor site morbidity was observed in any patient.

Scalp reconstruction has many goals. Restoring the bony contour, coverage of the defect, and return of soft tissue thickness are just a few. The new tissue must also be able to withstand the shear forces that may be applied to it in the future, heal in a timely fashion to allow adjuvant treatments to begin, and withstand future radiation or trauma.^7^^,^^8^ If postoperative radiation therapy is necessary, a free tissue flap will be less likely to necrose as it has a superior vascular supply when compared with non–free flaps and skin grafts.^9^ It is difficult to meet both structural and protective needs of the scalp with non–free tissue flaps in defects larger than 200 cm^2^. Risk of complications increases with local advancement and rotational flaps when multiple flaps have to be created to close the defect.^10^ From our cohort of patients with scalp defects larger than 50 cm^2^ who underwent reconstruction with free flap, local tissue transfer flap, and skin grafting, it appears that free tissue transfer with microvascular anastomosis is a viable primary choice for reconstruction of complex defects.

Multiple free flaps have been described, including latissimus dorsi,^11^^-^13 anterolateral thigh,^14^ radial forearm,^15^ rectus abdominus,^16^ omental,^17^ scapular,^18^ and serratus muscle flaps.^19^ Overall, the free latissimus dorsi muscle flap has become the preferred free flap for scalp reconstruction due to its large surface area, and long vascular pedicle.^20^ The latissimus dorsi muscle was chosen as a donor for the majority of our free flaps with either an immediate unmeshed split-thickness skin graft or delayed skin grafting to cover the flap. Although the muscle may be bulky initially, the flap typically thins over time due to muscle atrophy and ultimately replicates a normal scalp within a matter of months. There are no large free flaps available that can replace the hair-bearing characteristics of the normal scalp.^21^

Multiple technical considerations may improve outcomes for free flap scalp reconstruction. To improve flap survival, one must perform wide undermining of the galea around the pedicle tunnel if there is one and assure diligent postoperative patient positioning to prevent compression of flap pedicle. Using superficial temporal recipient vessels when possible and performing intramuscular pedicle dissection can increase pedicle length and eliminate the need for vein grafts. Covering the calvarial or cranioplasty sites with the proximal portion of the muscle flap and positioning the least reliable flap areas to minimize morbidity if partial flap loss were to occur can further improve recipient site outcomes.^22^

Scalp reconstruction is often further complicated by previous operative attempts at closure of the defect. Four of our free flap reconstruction patients had previously undergone an operation to close the scalp defect, either with skin grafting or non–free flap reconstruction, including our 2 free flap patients who required reoperations. In addition, traumatized and vascularly compromised tissue from radiation or infection increases the risk of local flap failure.^6^ Free tissue flaps have superior functional and esthetic outcome when compared with local tissue advancement flaps. Free flaps are often a better option for larger defects and for patients who have undergone previous reconstruction attempts or those requiring radiation therapy.^7^

Multiple algorithms have been proposed for a stepwise approach to the management of large scalp and forehead defects all with similar features.^1^^,^^20^^,^^23^^-^25 These can provide a framework and allow the surgeon to make generalized plans when approaching a scalp reconstruction patient. We advocate that free flap reconstruction be used as the first choice for complicated, large defects rather than attempting other options first. Our review of previously completed series of scalp reconstruction using free tissue flaps has shown similar results to our own,^1^^,^^5^^-^8^,^^20^^,^^24^^,^^26^^-^28 which supports the premise that free tissue transfer may be regarded as a primary method in larger scalp defect reconstruction.

## CONCLUSION

Free tissue transfer is a feasible option and we advocate for its use as the primary method for repairing large scalp defects. As techniques improve and technology continues to advance, immediate postoperative morbidity from microvascular free tissue transfer will likely decrease. A prospective study should be conducted to evaluate further differences in each group. A multicentered study would likely be necessary to obtain a signification number of patients to adequately evaluate each surgical option.

## Figures and Tables

**Figure 1:**
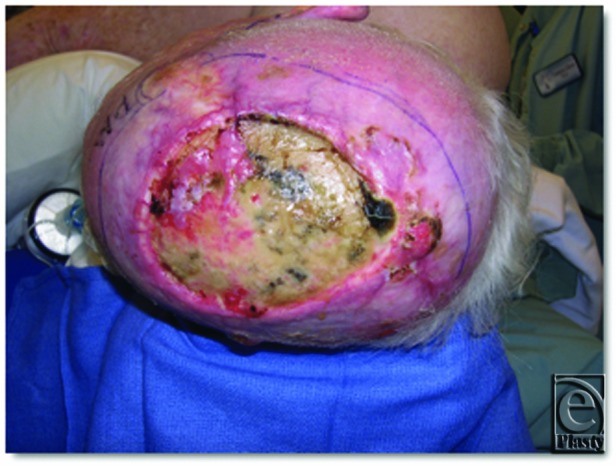
Patient with nonhealing wound following prior flap reconstruction and failure due to radiation.

**Figure 2:**
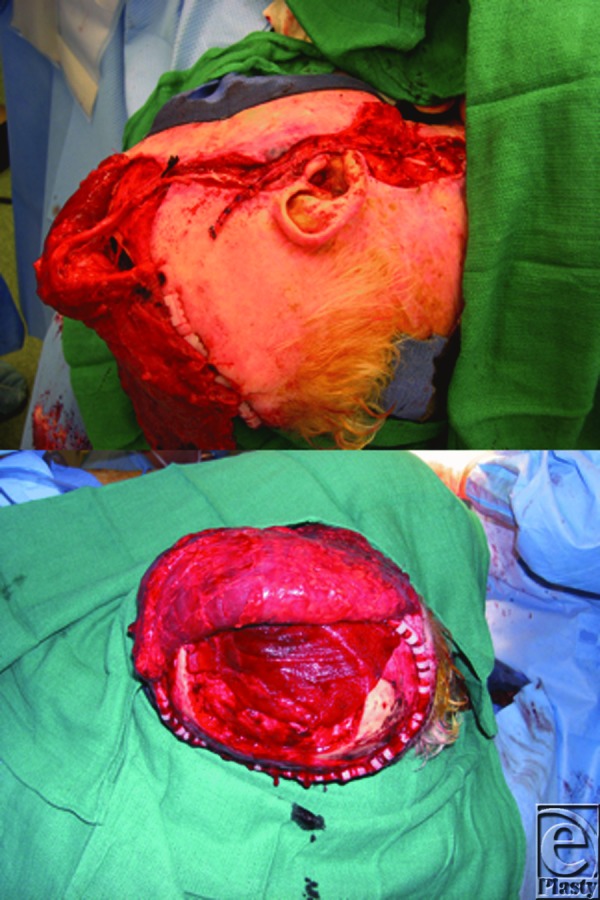
Intraoperative photograph during free latissimus dorsi muscle transfer with microvascular anastomosis.

**Figure 3:**
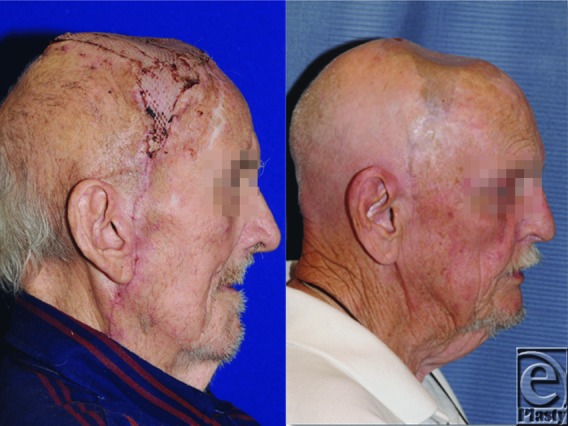
Postoperative free latissimus dorsi muscle transfer with skin graft.
